# Comparative Price Analysis of Biological Products for Treatment of Rheumatoid Arthritis

**DOI:** 10.3389/fphar.2018.01070

**Published:** 2018-09-20

**Authors:** Manoela Manova, Alexandra Savova, Maria Vasileva, Silvia Terezova, Maria Kamusheva, Daniela Grekova, Valentina Petkova, Guenka Petrova

**Affiliations:** ^1^Department of Organization and Economics of Pharmacy, Faculty of Pharmacy, Medical University of Sofia, Sofia, Bulgaria; ^2^National Council on Prices and Reimbursement of Medicinal Products, Sofia, Bulgaria; ^3^Department Economics of Trade, University of National and World Economy, Sofia, Bulgaria; ^4^Faculty of Pharmacy, Medical University of Plovdiv, Plovdiv, Bulgaria

**Keywords:** biological products, biosimilars, access, prices, rheumatoid arthritis

## Abstract

Biological products for treatment of rheumatoid arthritis usually are cost effective for healthcare systems in Europe, but they are huge financial burden due to the high number of patients and the significant budget impact. The expected saving from introduction on the market of biosimilars are significant and are linked to better access and affordability. The aim of this study was to conduct comparative price analysis of biological products for rheumatoid arthritis therapy among seventeen EU countries. The point of view is that of the Bulgarian pricing and reimbursement system and the chosen countries are those from external reference basket for prices comparison at manufacturing level. All authorized biological products by EMA with therapeutic indication rheumatoid arthritis were selected. The access for treatment is evaluated as the availability of the product on the market and the prices level. We assessed the availability of all trade names in the price lists of the observed countries. The prices data was obtained from the official web pages of the responsible institutions up to date December 2017. The results show that four out of all six INNs have authorized biosimilars in EMA. Despite its earlier authorization biosimilar adalimumab is not present in any of the price lists of countries. From all eighteen countries only in Lithuania and Estonia there were no published prices of any of the selected medicinal products. Countries with higher number of biosimilar prices are Spain and France. Differences in manufacturers’ prices of reference biological products in selected countries in comparison with the lowest manufacturer price are higher with 22 to 69% while the retail prices between 62 and 95%. Differences are mostly notable for rituximab, and less notable for tocilizumab. Manufacturers’ and retail prices of biosimilar products were established only for three INNs (etanercept, rituximab, and infliximab). Manufacturers’ prices differ between 26 and 75%, while retail prices differ between 40 and 92% for biosimilars. Comparison of the differences between manufacturer prices of reference biological product and biosimilars shows 36% difference for etanercept, 39% for rituximab, and 31% for infliximab, while at retail level the differences are 11, 86, and 143%, respectively. The limitation of the study is that the prices are the official ones without discounts due to confidentiality and the real prices may be lower. The second limitation is that the methodology for pricing differs in the countries and this could also influence the prices on both levels (manufacturer and retail). Introduction of biosimilars on the national markets led to significant decrease in reimbursed prices paid by public funds and thus might benefit the patients’ access to biological therapy. The decrease of prices after biosimilars entrance on the market is not as notable as for commodity generics.

## Introduction

Rheumatoid arthritis (RA) is the most frequent chronic, systemic autoimmune inflammatory disease that causes systemic pain, swelling, and destruction of the joints. The prevalence is around 0.5–1% of the population worldwide (Kobelt and Kasteng, 2009). The etiology may be different, but RA is often classified as autoimmune disease and has a genetic predisposition. The disease leads to functional disability, worsening in the quality of life and premature mortality (Chen et al., 2006; Aletaha et al., 2010). The life expectancy is significantly decreased with approximately 4 years in men, while in women with 10 years. The mortality is high especially in patients with early loss of mobility, those with acute episodes and patients with co-morbidities (Scott et al., 2010).

Over the last 20 years a significant improvement in the therapy for RA was observed with the introduction of disease modifying drugs (DMARDs) especially the biological DMARD (bDMARDs), which achieves the basic goal of the RA treatment – clinical remission (Smolen et al., 2007; Felson et al., 2011). The advantages of bDMARDs are their better therapeutic response, improvement in health-related quality of life and mobility, reduced disability and mortality (Chen et al., 2006; Jönsson et al., 2008; Smolen et al., 2017). The most prescribed bDMARDs are TNF-α inhibitors, which can control the inflammation, and to prevent or delay the bone erosion (Deighton and Hyrich, 2008). There is a trend toward increased use of bDMARDs, but variations through countries are still observed and the access therefore varies.

The use of biological products in countries from Central and East Europe is lower than that in Western Europe and there is a link between the cost for biological therapy and increased healthcare expenditures (Pease et al., 2011; Hoebert et al., 2012). The results are confirmed by other studies, which reveal an inequality and limited access in countries with lower income and dependence from the reimbursement policy for biological treatment (Putrik et al., 2014). The treatment of RA represents a serious economic burden and the limited access to therapy represents a serious social burden (Furneri et al., 2012). Some cost of illness studies show that the biggest share in direct costs for RA is associated with hospitalization due to the disease, like other social important diseases (Dimitrova et al., 2015). The indirect costs should be also considered in assessing the economic burden of RA as the days absent from work may vary between 2 and 30 days per year and indirect costs exceed the direct costs (Cooper, 2000). Estimation in total cost of RA from societal point of view in Europe and United States show around 45 billion euro in Europe and 41 billion euro in the United States (Lundkvist et al., 2008). In some countries the biological products for treatment of RA have a leading market share in outpatient expenditures.

There are evidences that biological products are acceptable for the reimbursement systems in terms of cost-effectiveness but they still pose a huge financial burden to healthcare systems due to the high number of patients and the significant budget impact (Chiou et al., 2004). Results from systematic review show that in patients who had failed synthetic DMARD (sDMARD) monotherapy, all of the comparisons found biological combination therapy to be cost effective (van der Velde et al., 2011). A study for the access to biological RA treatment in CEEC shows that the reasons for limited access are complex, and depend not only on economic factors, but also on the treatment guidelines, administrative hurdles and limited access to specialists (Orlewska et al., 2011). Even if Europe appears as a leader for the biosimilar market, accounting for 80% of global spending on these products, little information was available about biosimilar pricing and reimbursement policies. Prices control and biosimilar policy are crucial for the decrease in the reimbursed cost and in many countries appears to be a leading cost containment measure. The estimated savings from the introduction of biosimilars in RA may lead to an increase in the number of patients with access to treatment and to resolution of the inequalities in different countries (Gulácsi et al., 2015). The uptake of biosimilars in the market is limited not only due to the lower price erosion in comparison with generic products but also due to various factors, such as safety, manufacturing, entry barriers, physician acceptance etc., (Blackstone and Fuhr, 2012; Blackstone and Joseph, 2013; Dörner et al., 2013). The potential of biosimilars as cost containment tool can be reached after overcoming the barriers for market access through complex measures (Farfan-Portet et al., 2014; Moorkens et al., 2016; Rémuzat et al., 2017).

The aim of this study was to conduct comparative price analysis of biological products for RA therapy among seventeen EU countries. We explored the date of marketing authorization of biological and biosimilars with therapeutic indication RA (by INN) by EMA and compared manufacturer and retail prices. The main study questions were: if all authorized product have approved prices in all countries under consideration:

1. What is the price difference between the reference biological products (RBP) and biosimilars?
2. What is the difference between manufacturer and retail prices?
3. What are the differences at national level?

## Materials and Methods

The selection of the medicinal products is based on the therapeutic indication RA and only the biologic therapy was considered and the selected INNs are those available on Bulgarian market. First all authorized RBP (meaning originators) by EMA with therapeutic indication RA were extracted from the website with the date of initial marketing authorization. Six INN (adalimumab, etanercept, rituximab, tocilizumab, golimumab, and infliximab) were selected for study. Then authorized biosimilars were identified with the date of their marketing authorization by EMA.

We assessed the availability of all trade names and dosage forms in the price lists of all observed countries. The data was obtained from the official web pages of the responsible pricing institutions in the countries and the prices were compared in December 2017. We compared the officially published manufacturer price, and where were available the retail prices. If the retail price was not published we calculate it based on the respective regulation in countries if applicable. We determined pharmaceutical presentations, which were based on strength, pharmaceutical form and pack size.

Criteria for the selection of countries were the legislative basis in Bulgaria and the number of countries in the external reference basket for prices comparison at the manufacturing level. The references basket comprise of 18 selected EU countries – Bulgaria (BG), Romania (RO), Greece (GR), France (FR), Latvia (LT), Slovakia (SK), Lithuania (LI), Portugal (PT), Italy (IT), Slovenia (SI), Spain (ES), Belgium (BE), the Czechia (CZ), Poland (PL), Hungary (HU), Denmark (DK), Finland (FI), and Estonia (EE). The choice of the comparator medicines for the analysis is based on a list of medication made by the Bulgarian Health authorities and therefore the choice of reference countries is based on the countries with the lowest publicly known prices. All included countries have specific regulation for prices of the medicinal products. The different methodology for pricing in selected countries influences the different price level.

For the measurement and prices comparison we selected the manufacturer price per pack and where available officially published data we also compared the retail prices per pack, incl. value-added tax (VAT) for both. The prices of equivalent pack sizes were compared. All prices were expressed in Euro. The exchange rates of the national banks for the countries out of Eurozone were considered at the end of 2017. For Bulgaria (BG) 1Euro = 0,95 BGN; for Romania (RO) 1 Euro = 4,42 RON; for Hungary (HU) 1 Euro = 302.62 HUF; for Poland and Denmark the exchange rate is based on the average monthly value for December 2017.

## Results

Four out of all six INNs have authorized biosimilars in EMA. Logically those INNs with earlier issued marketing authorization (1998 – rituximab, 1999 – infliximab, 2000 – etanercept and 2003 – adalimumab) should be those with biosimilars available in the European market. The availability of reference biologic product (RBP) and biosimilars is presented in **Supplementary Table 1**. We found that despite its earlier authorization biosimilar adalimumab is not present in any of the price lists of countries under consideration pointing out that this might be due to data exclusivity. Biosimilar rituximab is present in eight of the countries under consideration and etanercept in nine.

From all eighteen countries only in Lithuania and Estonia there were no published prices of any of the selected medicinal products – **Supplementary Table 2**. A huge variability of price information is noted among the countries under consideration. Manufacturer prices of original etanercept were found from 13 to 15 countries depending on the pharmaceutical presentation, and for biosimilar from 3 to 9 countries possess manufacturer prices, while retail prices are less than that. For some trade names of biosimilar rituximab manufacturing prices are available in seven of the countries, but only four published retail prices. The other two biosimilar rituximab are present only in two countries as settled prices. Biosimilar infliximab prices were available in almost all countries except Lithuania and Estonia, but in France, Slovenia, Belgium, and Poland only manufacturer prices were available. Infliximab is also notable with less manufacturer (*n* = 5) and retail prices (*n* = 1) of originator than of biosimilar. Countries with higher number of biosimilar prices are Spain (*n* = 14) and France (*n* = 12). Lithuania and Estonia are only two countries in which prices of any biosimilar were not found. Latvia, Slovakia, and Portugal were having prices only for biosimilar infliximab. Tocilizumab and golimumab do not have biosimilars and RBP have manufacturing prices from 11 out of 15 and from 9 out of 13 retail prices, out of 17 countries (**Supplementary Table 2**).

**FIGURE 1:**
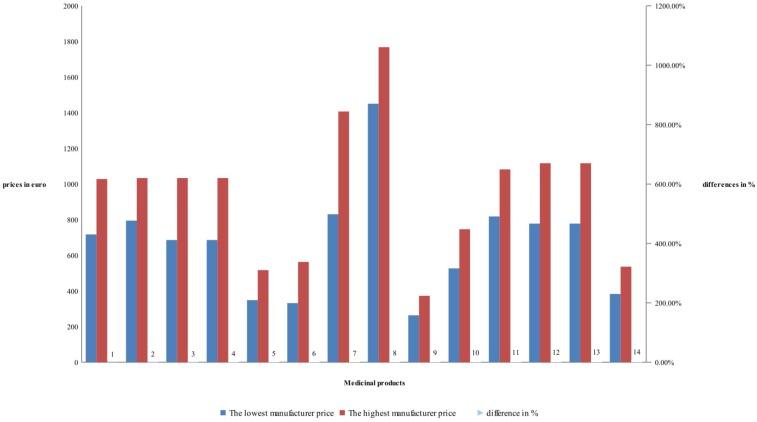
Differences in manufacturing prices of reference biological products.

**FIGURE 2:**
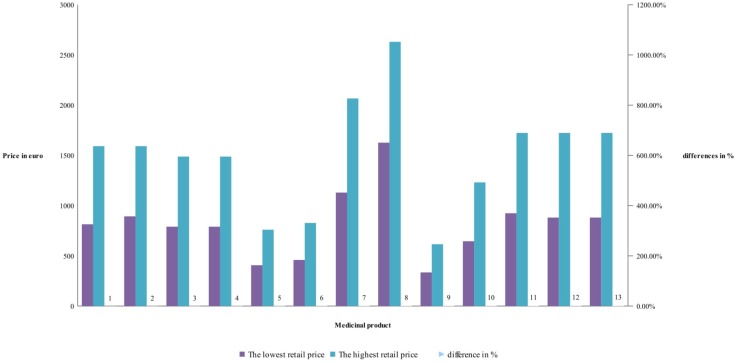
Differences in retail prices of reference biological products.

Detailed price comparison for manufacturers’ and retail price of RBP is shown on **Figures 1**, **2**. Prices comparison is based on the differences between the lowest price and the highest price. Differences in manufacturers’ prices are varying between 22 and 69% while the retail prices between 62 and 95%. Differences are mostly notable for rituximab RBP, and less notable for tocilizumab – (**Figure 1**).

Differences between retail prices of RBP are between 62 and 95%, but mostly around 85% difference between the lowest and highest retail price of RBP – (**Figure 2**). Lowest retail prices possess etanercept. Products with biosimilar have high differences. The difference in retail prices is due also to the methodology in the countries.

**FIGURE 3:**
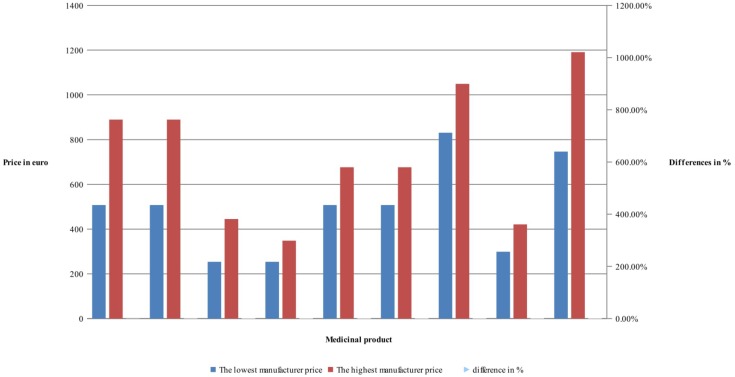
Differences in manufacturers’ prices of biosimilar.

**FIGURE 4:**
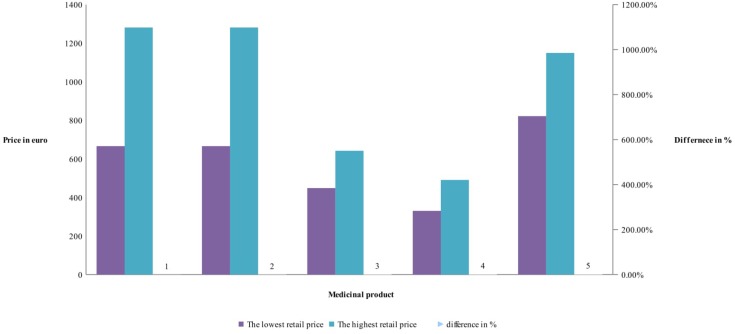
Differences in retail prices of biosimilar.

Manufacturers’ and retail prices of biosimilar products were established only for three INNs (etanercept, rituximab, and infliximab). Manufacturers’ prices differ between 26 and 75%, while retail prices differ between 40 and 92% for biosimilars. Manufacturers prices of biosimilar etanercept were established in 9 out of 17 observed countries, and 3 countries were having more than one biosimilar. Manufacturing prices of rituximab were established in 7 of the observed countries (**Figures 3**, **4**).

**FIGURE 5:**
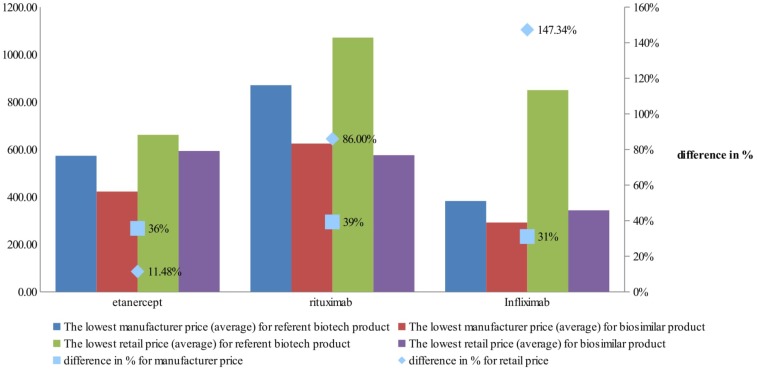
Difference in manufacturers prices between reference biological product and biosimilar.

Comparison of the differences between manufacturer prices of RMP and biosimilars shows 36% difference for etanercept, 39% for rituximab, and 31% for infliximab, while at retail level the differences are 11, 86, and 143%, respectively (**Figure 5**). As was noted the RBP of infliximab was present in limited number of countries.

Country comparison between the prices of RBP and biosimilar at manufacturing and retail level could be made only for etanercept. The higher difference is in Italy where the price of biosimilar is 45% less with two authorized biosimilars and the lower difference is in Spain (10%) with six biosimilars. In the other countries the retail prices differ between 15 and 25% lower for biosimilars. For rituximab only at retail level could be made a price comparison. Again, Spain has high number of biosimilars and price difference with 15% at retail level, while Hungary has six biosimilars with 43% price difference at retail level.

## Discussion

Prices of biosimilars and their RBP have been studied from other European authors providing general overview of the pricing approaches, patients’ access and policies (Moorkens et al., 2017; Rémuzat et al., 2017). This study adds more to the reference pricing systems influence in 17 European countries on the prices of biologicals for one disease that is the RA. We choose Bulgaria as reference country due to its low pricing policy and compare prices with its reference basket of 18 countries countries. Results show that manufacturer prices in Bulgaria are really among the lowest one during the moment of observation, but retail prices are probably influenced by additional factors such as the VAT and the mark up policies.

In contrast with other studies we focused not to price level but mostly to price differences of the products for RA therapy. One study (Moorkens et al., 2017) pointed out that some countries require the first and then every next biosimilar to be less priced with at least 30% than the reference biological product. We found that the difference between biosimilars and RBP at manufacturing level is between 36 and 39% that could be commented as successful measure for price decrease, which is more than in Western Europe. Despite the decrease in prices when first biosimilar appears we consider that still there are many other obstacles in front the significant price decrease in all the countries. The fact that there is a difference between manufacturers and retail prices reaching almost 90% in some countries could be commented as the evidence that biosimilars influence significantly the prices of RBP, but the huge differences between the products could be due to other measures. Margins and taxes are different in the different countries, resulting in varying price differentials along the pharmaceutical market. Those differences cannot be attributed only to the manufacturer, but also to national policies, as is the tender policy for hospitals. Therefore, the comparison in prices should be done at manufacturer level because retail price is influenced by other factors (regulation, policy, and pricing methodology).

Access to biosimilars is not equal in all the countries in consideration. Adalimumab was not having a biosimilar price in any of the observed countries that might be due to patent protection or data exclusivity clauses. This fact could be commented as efforts from the originator companies to extend the patent protection as longer as possible (Derbyshire, 2015).

Infliximab is the other example for the influence of the manufacturer policy on the access to biosimilars. The RBP was present at five countries at manufacturing level in only one country at retail level, while the two biosimilars were present in almost all the countries (*n* = 14). For the other INNs where biosimilar is available RPB possess prices in many more countries than the biosimilars that could be commented as delay of the biosimilars to the national markets.

Tuna et al. performed a systematic review in order to investigate the price difference between biotechnological reference products and biosimilars. The authors revealed that this difference varies across the European countries between 0.51 and -38%, which is similar with the results for the United States and Turkey. Despite the lower differences in comparison with the conventional and generic drugs, the expected cost savings are more than 10 billion dollars (Tuna and Caliskan, 2017).

A comparison of the prices of anti-TNF biosimilars between Canada and European countries showed that the differences are statistically significant as the biosimilar price discount is greater in Canada: 36 vs. 22% in Northern Europe and 18% in Southern Europe (Gauthier et al., 2017).

According to literature data the differences between the price of biosimilars and their RBP is between 15 and 30%. Whereas this difference for generics and originator drugs may be up to 80% (Declerck and Simoens, 2012; Vogler et al., 2016). Haustein et al. (2012) concluded that the use of biosimilars could significantly decrease the healthcare expenditure on biological medicines in EU national markets (2007–2020) of France, Germany, Italy, Poland, Romania, Spain, Sweden, and United Kingdom. The greatest savings are expected to occur as a result of entering biosimilar monoclonal antibodies on the market – between 1.8 billion to 20.4 billion euro. Utilization of bDMARDs varies across the countries as it is very low in Poland 1.3%, Bulgaria 2.6%, Romania 4.1%, and the Czechia 4.2%. Availabilities of cheaper therapies such as biosimilars could significantly improve patients’ access to biological antirheumatic therapy (Dörner et al., 2016).

The expected price erosion from biosimilars in the United Kingdom and Germany is around 35%, which is far below that for generics (Farfan-Portet et al., 2014).

Gulácsi et al. (2015) commented that introduction of biosimilars could be associated with providing of cost effective alternatives to the expensive reference biotechnological medicinal products and our study could confirm such a comment. Biosimilars have not proven cost effective data on their drug as they used the reference product data to show cost effectiveness.

The market share of biosimilars has been increasing in the recent years. A significant difference is observed among the countries regarding biological medicines price (around 20–40%) and the peak of biosimilar penetration (10–35%). Similarity between the price differences for biosimilars market and generics one is not revealed as the price differentials are smaller for biosimilars and reference products (Rovira et al., 2013).

Each country implements different incentive policies regarding biosimilars, which determines the variety in the number of biosimilars available on the market (Rémuzat et al., 2017). As study limitation we can pointed out that biosimilar cannot be compared to generic as for example the cost of goods are far higher. The study is also focused mainly on price differences between the reference for Bulgaria countries and not on the policy approaches. The prices and reimbursement analysis focused on the biologicals, which are listed in the Bulgarian Positive drug list and therefore some biologicals are not included in the analysis. We recognize that the pricing and reimbursement in Bulgaria is based on the reference basket of 18 EU countries, which consists mostly in low pricing countries (Kawalec et al., 2017). Further analysis should be done for policy influence on the price differences.

## Conclusion

Introduction of biosimilars on the national markets led to significant decrease in reimbursed prices paid by public funds and thus might benefit the patients’ access to biological therapy. The decrease of prices after biosimilars entrance on the market is not as notable as for commodity generics. This is expected and can be explained with the complexity of the biological molecules and the higher expenditures for manufacturing compared with the generics. The potential of biosimilars for reducing prices and expenditures is not so notable in the first years after market entrance. Further analysis is needed to evaluate the access and availability of biological treatment after introduction of more biosimilars and after some time the products are on the market. The external reference pricing applicable in most European countries may lead to smaller differences in manufacturer prices, but the retail price differ in the countries due to differences national policies.

## Author Contributions

GP and MM defined the goal of the study, its conception and design. MM, AS, MV, and ST collected the data. MM coordinated the project. MM, AS, MK, DG, VP, and GP carried out the interpretation of data and prepared the draft of the manuscript. All authors contributed to editing and approving the final version of the manuscript.

## Conflict of Interest Statement

The authors declare that the research was conducted in the absence of any commercial or financial relationships that could be construed as a potential conflict of interest.
