# Supplementary material for: Comparative Price Analysis of Biological Products for Treatment of Rheumatoid Arthritis
Source: Front Pharmacol. 2018 Sep 20;9:1070. doi: 10.3389/fphar.2018.01070 (PMC6158404; doi:10.3389/fphar.2018.01070)
Supplement: Supplementary file 1 [file Table_1.DOCX]

**Table 1. Availability of biologic products for treatment of RA**

| INN | Product | BG | RO | GR | FR | LT | SK | LV | PT | IT | SI | ES | BE | CZ | PL | HU | DK | FI | EE |
| --- | --- | --- | --- | --- | --- | --- | --- | --- | --- | --- | --- | --- | --- | --- | --- | --- | --- | --- | --- |
| adalimumab | RBP | + | + | - | + | + | + | - | + | + | + | + | + | + | + | + | - | + | - |
|  | biosimlar | - | - | - | - | - | - | - | - | - | - | - | - | - | - | - | - | - | - |
| etanercept | RBP | + | + | - | + | + | + | + | + | + | + | + | + | + | + | + | + | + | - |
|  | biosimlar | - | + | + | + | - | - | - | - | + | - | + | + | + | + | - | + | - | - |
| rituximab | RBP | + | + | + | + | + | + | - | - | + | + | + | + | + | + | + | + | - | - |
|  | biosimlar | + | - | - | + | - | - | - | - | - | + | + | + | + | + | + | - | - | - |
| golimumab | RBP | + | + | - | + | + | + | + | + | + | + | + | + | + | + | + | + | + | - |
| tocilizumab | RBP | + | + | - | + | + | + | + | + | + | + | + | + | + | + | + | + | + | - |
| infliximab | RBP | - | - | - | - | - | + | - | - | + | - | + | - | - | + | + | - | - | - |
|  | biosimlar | + | + | - | + | + | + | + | + | + | + | + | + | + | + | + | + | + | - |
