# Supplementary material for: Comparative Price Analysis of Biological Products for Treatment of Rheumatoid Arthritis
Source: Front Pharmacol. 2018 Sep 20;9:1070. doi: 10.3389/fphar.2018.01070 (PMC6158404; doi:10.3389/fphar.2018.01070)
Supplement: Supplementary file 2 [file Table_2.DOCX]

**Table 2. Available price information in the observed countries**

| **INN** | **pharmaceutical presentation** | **Available information for manufacturer prices** | **Available information for retail prices** |
| --- | --- | --- | --- |
| Adalimumab (RBP) | 40 mg/0,8 ml solution for injection x 2 pre-filled syringe | ***11 countries***  *RO, FR, PT, IT, SI, ES, BE, CZ, PL, HU, FI* | ***9 countries***  *RO, FR, PT, IT, ES, BE, CZ, HU, FI* |
| Adalimumab (RBP) | 40 mg/0.8 ml Solution for injection x 2 carton pack | ***11 countries***  *FR, LV, SK, PT, IT, SI, ES, CZ, PL, DK, HU* | ***9 countries***  *FR, LV, SK, PT, IT, CZ, PL, DK, HU* |
| Adalimumab (biosimilar 1) | 40 mg/0,8 ml solution for injection x 2 pre-filled syr | No | No |
| Adalimumab (biosimilar 2) | 40 mg/0,8 ml solution for injection x 2 pre-filled syr | No | No |
| Adalimumab (biosimilar 3) | 40 mg/0,8 ml solution for injection x 2 pre-filled syringe | No | No |
| Adalimumab (biosimilar 4) | 40 mg/0,8 ml solution for injection x 2 pre-filled syr | No | No |
| Etanercept (RBP) | 50 mg/ml-1 ml solution for injection x 4 pre-filled pens +4 swabs | ***13 countries***  *RO, EL, LV, SK, PT, SI, ES, BE, CZ, PL, HU, DK, FI* | ***11 countries***  *RO, EL, LV, SK, PT, ES, BE, CZ, HU, DK, FI* |
| Etanercept (RBP) | 50 mg/ml-1 ml solution for injection x 4 pre-filled syringes +4 swabs | ***14*** ***countries***  *RO, EL, FR, LV, SK, PT, SI, ES, BE, CZ, PL, HU, DK, FI* | ***12 countries***  *RO, EL, FR, LV, SK, PT, ES, BE, CZ, HU, DK, FI* |
| Etanercept (RBP) | 25 mg powder and solvent solution for injection x 4 vials + 4 prefilled syr | ***15 countries***  *RO, EL, FR, LV, SK, PT, IT, SI, ES, BE, CZ, PL, HU, DK, FI* | ***13*** ***countries***  *RO, EL, FR, LV, SK, PT, IT, ES, BE, CZ, HU, DK, FI* |
| Etanercept (biosimilar 1) | 50 mg/ml-1 ml solution for injection x 4 pre-filled pens | ***9 countries***  RO, EL, FR, IT, ES, BE, CZ, PL, DK | ***8 countries***  RO, EL, FR, IT, ES, BE, CZ, DK |
| Etanercept (biosimilar 1) | 50 mg/ml-1 ml solution for injection x 4 pre-filled syr | ***9 countries***  RO, EL, FR, IT, ES, BE, CZ, PL, DK | ***8 countries***  RO, EL, FR, IT, ES, BE, CZ, DK |
| Etanercept (biosimilar 1) | 25 mg/0,51 ml solution for injection x 4 pre-filled syr | ***8 countries***  RO, EL, FR, IT, ES, CZ, PL, DK | 7 countries  RO, EL, FR, IT, ES, CZ, DK |
| Etanercept (biosimilar 2) | 50 mg/ml - 0.5 ml solution for injection x 4 pre-filled syr | ***3 countries***  FR, ES, PL | ***1 countries***  ES |
| Etanercept (biosimilar 2) | 50 mg/ml - 1 ml solution for injection x 4 pre-filled syr | ***3 countries***  FR, ES, PL | ***1 countries***  ES |
| Etanercept (biosimilar 2) | 50 mg/ml - 1 ml solution for injrctionx 4 pre-filled pens | ***3 countries***  FR, ES, PL | ***1 countries***  ES |
| Etanercept (biosimilar 3) | 25 mg Powder and solvent for sol for injx 4 vials + 4 pref syr | No | No |
| Etanercept (biosimilar 3) | 25 mg/0,5 ml solution for injection x 4 pre-filled syr | No | No |
| Etanercept (biosimilar 3) | 50 mg/1 ml solution for injection x 4 pre-filled syr | No | No |
| Etanercept (biosimilar 3) | 50 mg/1 ml sol for injection | No | No |
| Rituximab (RBP) | 100mg/10 ml Concentrate for solution for infusion x 2 vials | ***13 countries***  *RO, EL, FR, LV, SK, IT, SI, ES, BE, CZ, PL, HU, DK* | ***9 countries***  *RO, EL, LV, SK, IT, ES, CZ, HU, DK* |
| Rituximab (RBP) | 500 mg/50 ml concentrate for sol for infusion | ***13 countries***  *RO, EL, FR, LV, SK, IT, SI, ES, BE, CZ, PL, HU, DK* | ***9 countries***  *RO, EL, LV, SK, IT, ES, CZ, HU, DK* |
| Rituximab (RBP) | Solution for injection, 1400 mg/11.7 ml x 1 | ***13 countries***  *RO, EL, FR, LV, SK, IT, SI, ES, BE, CZ, PL, HU, DK* | ***9 countries***  *RO, EL, LV, SK, IT, ES, CZ, HU, DK* |
| Rituximab (biosimilar 1) | 100mg/10 ml conc for sol for inf x 2 vials |  |  |
| Rituximab (biosimilar 1) | 500 mg/50 ml concentrate for solution for inf x 1 vial | ***2 countries***  *FR, ES* | ***1 country***  *ES* |
| Rituximab (biosimilar 2) | 100mg/10 ml Concentrate for solution for infusion x 2 vials | ***1 country***  ES | ***1 country***  ES |
| Rituximab (biosimilar 2) | 500 mg/50 ml concentrate for solution for infusion x 1 vial | ***1 country***  ES | ***1 country***  ES |
| Rituximab (biosimilar 3) | 100mg/10 ml Concentrate for solution for infusion x 2 vials | ***3 countries***  *FR, ES, HU* | ***2 countries***  ES, HU |
| Rituximab (biosimilar 3) | 500 mg/50 ml concentrate for solution for infusion x 1 vial | ***6 countries***  *FR, ES, SI, HU, BE, CZ* | ***3 countries***  ES, HU, CZ |
| Tocilizumab (RBP) | 20 mg/ml - 10 ml Concentrate for solution for infusion x 1 vial | ***12 countries***  *RO, EL, FR, SK, LT, PT, IT, SI, ES, PL, HU, DK* | ***9 countries***  *RO, EL, SK, PT, IT, ES, BE, HU, DK* |
| Tocilizumab (RBP) | 20 mg/ml - 20 ml concentrate for solution for inf x 1 vial | ***11 countries***  *RO, EL, FR, SK, LT, PT, IT, SI, PL, HU, DK* | ***8 countries***  *RO, EL, SK, PT, IT, BE, HU, DK* |
| Tocilizumab (RBP) | 162 mg Solution for injection in pre-filled syringe x 4 pre-filled syringes | ***15 countries***  *EL, FR, LV, SK, PT, IT, SI, ES, PL, HU, DK, BE, CZ, FI* | ***12 countries***  *EL, LV, SK, PT, IT, ES, HU, DK, FR, BE, CZ, FI* |
| Golimumab (RBP) | 50 mg/0,5 ml solution for injection x 1 pre-filled syringe | ***11 countries***  *RO, FR, SK, PT, IT, ES, BE, CZ, HU, DK, FI* | ***11 countries***  *RO, FR, SK, PT, IT, ES, BE, CZ, HU, DK, FI* |
| Golimumab (RBP) | 50 mg/0,5 ml solution for injection x 1 pre-filled pen | ***15 countries***  *RO, EL, FR, LV, SK, PT, IT, ES, BE, CZ, HU, DK, FI, SI, PL* | ***13 countries***  *RO, EL, FR, LV, SK, PT, IT, ES, BE, CZ, HU, DK, FI* |
| Infliximab (RBP) | remicade 100mg powder for conc for sol for inf vials x 1 | ***5 countries***  SK, IT, ES, PL, HU | ***1 country***  IT |
| Infliximab – biosimilar 1 | Powder for concentrate for sol for inf 100 mg x 1 | ***14 countries***  *RO, EL, FR, LV, SK, PT, IT, SI, ES, BE, CZ, PL, HU, DK* | ***10 countries***  *RO, EL, LV, SK, PT, IT, ES,CZ, HU, DK,* |
| Infliximab – biosimilar 2 | Powder for concentrate for solution for inf 100 mg x 1 | ***14 countries***  *RO, FR, LV, SK, PT, IT, SI, ES, BE, CZ, PL, HU, DK, FI* | ***10 countries***  *RO, EL, LV, SK, PT, IT, ES,CZ, HU, DK, FI* |
